# Patients with Tetralogy of Fallot have lower systolic KE in the left ventricle and higher diastolic KE in the right ventricle

**DOI:** 10.1186/1532-429X-18-S1-P184

**Published:** 2016-01-27

**Authors:** Pia Sjöberg, Einar Heiberg, Håkan Arheden, Ronny Gustafsson, Shahab Nozohoor, Marcus Carlsson

**Affiliations:** 1 Dept of Clinical Physiology, Lund University, Lund, Sweden; 2 Department of Cardiothoracic Surgery, Lund University, Lund, Sweden

## Background

There is a persisting debate regarding indications and timing of pulmonary valve replacement in patients with pulmonary insufficiency after repair of Tetralogy of Fallot (ToF). The status of right ventricular function is important is this decision but remains elusive. 4D-flow CMR enables quantification of ventricular kinetic energy (KE) which may provide improved understanding of the pathophysiology of the dilated ventricle with impaired function. In this study we wanted to quantify ventricular KE in patients with pulmonary insufficiency after repair of ToF.

## Methods

Nine patients with pulmonary insufficiency after repair of ToF (3 females, median age 22 years, range 18-51) underwent cardiovascular magnetic resonance (CMR) with a 1.5 T Philips scanner including four-dimensional phase-contrast flow sequence. Eight healthy volunteers (2 females, median age 26, range 23-36) were used as controls. Ventricular segmentation was performed in cine ssfp images, 30 time frames per cardiac cycle covering the heart and imported to the 4D flow dataset. Ventricular KE calculated as KE=½mv² was summed over all voxels inside the right and left ventricle and calculated for each time frame.

## Results

**Figure 1:**
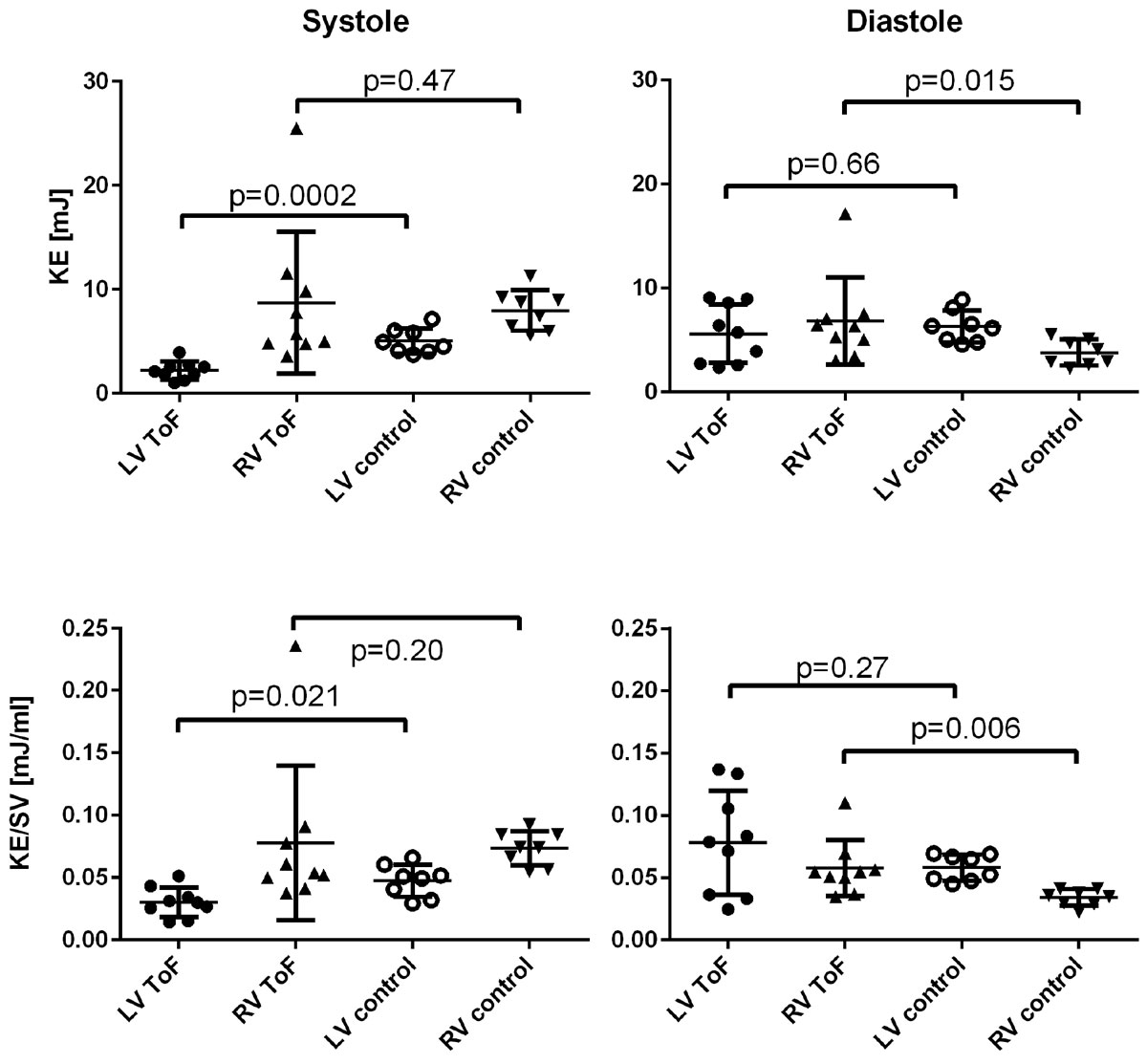
**Comparison of kinetic energy (KE) (top) and KE indexed for ventricular stroke volume (SV) (bottom) between ToF patients and controls in both left ventricle (LV) and right ventricle (RV)**. Bar and whiskers show mean ± SD.

Peak systolic KE, as well as KE indexed for the stroke volume (SV) of the ventricle was lower in the left ventricle of ToF patients than in controls. Peak diastolic KE was higher in the right ventricle of patients than in controls, also when KE was indexed for SV, Figure 1. Diastolic KE was mainly located in the AV-valve inflow to the ventricles in controls but was also seen in the regurgitant volume in RV of ToF patients.

## Conclusions

ToF patients have decreased left ventricular systolic KE compared to controls, indicating left ventricular affection in ToF patients, possibly through septal dyssynchrony. During diastole, right ventricular KE is increased compared to controls, most likely caused by the pulmonary regurgitation. Thus, 4D-flow MRI offers new insights in ToF physiology and inter-ventricular interaction.

